# Fostering student and faculty scholarship in an accelerated three-year medical school

**DOI:** 10.12688/mep.19142.1

**Published:** 2022-06-23

**Authors:** Lora J. Kasselman, Gladys Ayala, Steven Shelov, Jeannine Nonaillada

**Affiliations:** 1 Department of Foundations of Medicine, NYU Long Island School of Medicine, Mineola, New York, 11501, USA; 2 Department of Medicine, NYU Long Island School of Medicine, Mineola, New York, 11501, USA; 3 Department of Pediatrics, NYU Long Island School of Medicine, Mineola, New York, 11501, USA; 4 Department of Rehabilitation Medicine, NYU Long Island School of Medicine, Mineola, New York, 11501, USA

**Keywords:** research competency, faculty development, scholarly productivity, undergraduate medical education (UME)

## Abstract

**Background: **In acknowledgement of the importance of research competency in academic medicine, an extracurricular student research program and faculty development researcher pathway was developed to promote scholarly productivity at New York University (NYU) Long Island School of Medicine (LISOM), a three-year accelerated Liaison Committee on Medical Education (LCME)-accredited medical school. The aim was to enhance medical students’ and faculty scholarly productivity, by creating new training programs targeting research skills and academic collaboration. Impact was assessed by initial review of the extracurricular student research program and faculty development researcher pathway.

**Methods: **Electronic surveys via Google were sent out to all current (n = 72) students on 9/20/2021 and the faculty identified based on their primary appointment to NYU LISOM in the learning management system on 9/17/2021 to determine participation in research, presentation of research findings, satisfaction with the program, and research opportunities for students.  Student scholarly productivity was tracked using PubMed, restricted to search years 2020 through 2022.  For the faculty development researcher pathway, publications were tracked for each participant before and after completion of the program, with pre- and post-completion dates ranging from 2012 through 2020.

**Results: **Student survey results (29 responses out of 72) indicated 28% of students were involved in research with institutional faculty and 59% were interested in starting a research project. Most students involved in extracurricular research were satisfied with their experience and eight students have publications with faculty.  For the faculty development researcher pathway, 35% of the participants increased publications after program graduation.

**Conclusions:** Outcomes from the student research program and faculty researcher pathway were positive regarding student research engagement and faculty scholarly productivity, though long-term outcomes are yet to be evaluated. Progress will be tracked as students continue through undergraduate and graduate medical education, and as both students and faculty progress throughout their career.

## Introduction

Competency in research is important for practicing physicians, enhancing critical evaluation of evidence and solving community-based health issues ^1, 2^. Early exposure to research fosters critical thinking, an essential enduring skill throughout the career ^3^. Conducting research during undergraduate medical education is a pressing need due to primary care physician-scientist shortages ^4^, and best practices in medicine depend upon clear understanding of evidence-based medicine ^3, 5^. Many medical schools provide opportunities for students to conduct research during dedicated curricular blocks, but these brief programs may not be enough to fully develop life-long research skills ^1^. New York University’s Long Island School of Medicine (NYU LISOM) is a three-year medical (MD) program, accredited by the Liaison Committee on Medical Education (LCME), focused on educating primary care physicians, with curricular emphasis on incorporating evidence-based medicine into clinical practice. Because NYU LISOM is an accelerated program, with no fourth year, there are limited opportunities for students to dedicate large blocks of time to research, though the inaugural class at NYU LISOM indicated that they were interested in clinically-oriented research opportunities with faculty. Despite these limitations, leadership at NYU LISOM instituted an extracurricular student research program fostering research collaborations between interested students and faculty.

Concurrent with inaugural class matriculation, clinical faculty at NYU LISOM experienced increased expectations of scholarship, like others in academic medicine ^6– 10^. This is challenging with clinical, teaching, and service expectations placed on faculty coupled with compressed student curricular time ^8^. Additionally, physicians may not be well-trained in research and need institutional support to get started ^11– 14^. While early exposure to scholarship is ideal, these skills can be learned during graduate or continuing medical education, promoting critical thinking and effective application of evidence-based medicine in clinical practice ^1– 3^. Like institutional support provided by leadership for the student research program, support was provided, incorporating a research arm into an already established longitudinal faculty development program.

This paper describes how academic leaders at our institution approached challenges with new programmatic research initiatives for students and faculty resulting in minimized barriers to research opportunities, new collaborations, and scholarly productivity. 

## Methods

### Ethical statement

The primary author filled out the NYU Langone Health quality improvement (QI) self-certification form and determined that this project is considered QI and not research involving human subjects. Because this is consistent with the federal regulations governing human subject research, no IRB review or oversight was needed per institutional policy and no written consent for voluntary surveys were required. An independent faculty member who was not involved in the study reviewed the form and confirmed that ethical approval was not required.

### Student research program

In early 2020, a faculty member was assigned as student research advisor to develop and lead an extracurricular medical student research program. Confidential online surveys were created in Google and sent via email to 237 faculty, identifying potential research opportunities for interested students. Survey questions were developed by faculty and administrators in the student research working group to address whether faculty had research opportunities available, the type of research available, and time commitment expected of students. Questions were evaluated for face and content validity via review by faculty and administrative members of the student research working group. All faculty affiliated with the school were identified using the school’s learning management system. No changes were made to the survey initially, though after one year of faculty-student research, questions were added to address faculty satisfaction with student researchers (extended data file 1) ^15^. Specifically, there were three questions added asking if the faculty had or was currently conducting research with students, their satisfaction with their research student(s), and any other comments related to their research experience(s) with students. Simultaneously, surveys created on Google were sent to all current students via email (n=72). Survey questions were developed by faculty and administrators in the student research working group to address whether students had interest in extracurricular research and informing students that participation in research requires mandatory ethics and compliance training. Questions were evaluated for face and content validity via review by the faculty and administrative members of the student research working group. No changes were made initially to the survey but after one year, questions were added which addressed student satisfaction with their research experience and the student research program in general (extended data file 2) ^15^. All surveys were collated using Google Forms.

Of the 237 faculty identified based on their primary appointment to NYU LISOM through our learning management site that were sent the survey, 15 responded in total. Despite the low response rate, 15 faculty were deemed an adequate number of available mentors for our 24 students per class. Following this, a dedicated web space was created on the learning management system where students could identify research opportunities and access ethics training modules. These research opportunities were designed to be extracurricular and voluntary. Additionally, promotion of research was intended to commence in the first year of medical school and potentially continue throughout students' time at NYU LISOM, including their graduate medical education, if they stayed within the institution, providing three to six years of research exposure. Research opportunities were periodically updated via annual faculty surveys.

Since the extracurricular medical student research program began in early 2020, it was heavily impacted by the COVID-19 pandemic. Based on our experience during the pandemic, both faculty and students reconsidered how research was conducted, e.g., favoring research conducted remotely versus onsite. This benefitted students, affording more remote opportunities including chart reviews, literature searches, and case reports. 

To further promote student research opportunities, a biannual extracurricular research workshop was held and is now an enduring activity. The student research working group, comprised of faculty and medical school administration, allocated a two-hour block outside of normal curricular hours, two times a year in order to accommodate students across all three years. The first workshop occurred in the fall of 2020 and we now have one workshop in the fall and another in late winter/early spring. The faculty leading the workshop is a PhD educator in the medical school with extensive teaching experience. The initial workshop was held in-person (with virtual workshops held during the COVID-19 pandemic) at the medical school in a very informal setting, with dinner provided for attendees. During the initial workshop, students were given guidance on identifying projects and mentors, as well as ethics training. There were networking sessions where students conducting extracurricular research shared their experiences with new students. The workshop also highlighted invited faculty looking for student researchers. Initially, these presentations were given as brief didactics but subsequently, it evolved into a round-table format where students and faculty could interact with each other freely based on areas of interest. Lastly, the workshop included educational content such as abstract writing and poster construction aligning with the institution’s Annual Hospital Research Day. This ensures students involved in research have necessary tools to submit their work to other venues and gain valuable public presentation skills. 

Presently, annual confidential online surveys are sent to all medical students (N=72 total; n=24 per class) obtaining feedback on students' satisfaction with the research program, satisfaction with faculty mentors, areas for improvement, and scholarly activity, if involved in research (extended data figure 2) ^15^. Additionally, a PubMed search is conducted monthly identifying medical students who have published with NYU LISOM faculty. The PubMed search includes each student’s first and last name in the author field and “Long Island School of Medicine” with the Boolean “AND” between fields. The search is currently set to run from 2020 through the current year. As described earlier, additional questions were added to the student research survey, after one year of the program, to address the level of satisfaction with research opportunities and faculty mentorship.

### Faculty development program in research

Since 2012, a longitudinal faculty development program has existed at our site, ( https://medli.nyu.edu/faculty/faculty-development-mentoring/faculty-scholars-program), beginning when the hospital was serving as clinical campus for another medical school prior to becoming NYU LISOM. Focused on enhancing educator proficiency, this 12-month curriculum consisted of traditional teaching and learning pedagogy while nurturing academic scholarship. Participants had to complete a capstone project demonstrating impact in implementing one educational curricular facet. Despite their efforts, research underpinnings in these projects were lacking, deflating any purposeful outcomes. Furthermore, from years 2012 to 2016, only one of these projects resulted in a peer-reviewed publication. 

In 2016, leadership changed the program curriculum with increased research content. Added classes included: formulating research questions, performing literature searches using databases, critically appraising literature, using reference citation software, navigating the institutional review board (IRB) and ethical concerns in research, constructing surveys, choosing research designs, analyzing and interpreting data, and writing for publication. As a result, participants’ scholarly achievements began to surface, including one peer-reviewed publication, one peer-reviewed meeting abstract publication, two peer-reviewed posters at national and/or regional meetings, and one national grant award. Although there was increased scholarly success with added research curricular content, based on participant feedback and advisement of the program director, it was decided that having all participants complete mandatory research was not optimal. Medical research is something done out of yearning or personal inquiry ^16^, and does not work best when required.

To reflect this, in 2020, this program was reformatted with two distinct curricular pathways: The Educator Pathway and the Researcher Pathway. The 12-month Educator Pathway consists of traditional teaching and learning pedagogy whereas the 18-month Researcher Pathway includes focused research classes, with six additional months allowing more time for carrying out independent research, such as submitting to the IRB, robust data collection, careful data analysis, and crafting written results ( Table 1). 

**Table 1.** Faculty educator and researcher development program curricula at New York University Long Island School of Medicine.

Educator Pathway (12 months)	Researcher Pathway (18 months)
Learning Theories	Learning Theories
	IRB Basics
	How to Conduct Literature Searchers and Critical Appraisal
Fostering Scholarship	Fostering Scholarship
Curriculum Development	Curriculum Development
	Reference Citation Software Management
Large Group Teaching	Large Group Teaching
	Sampling Strategies
	Levels of Variable Measurement and Survey Design
Problem Based Learning	Problem Based Learning
Innovative Technology and Instructional Design	Innovative Technology and Instructional Design
Small Group Teaching	Small Group Teaching
	Data Analysis and Interpretation
Using Simulation in Medical Education	Using Simulation in Medical Education
Clinical Teaching	Clinical Teaching
Effective Feedback	Effective Feedback
	How to Prepare a Poster for a Professional Meeting
Strategies of Remediation for Medical Learners	Strategies of Remediation for Medical Learners
Mentoring in Academic Medicine	Mentoring in Academic Medicine
	Writing for Manuscript Publication
	Grant Writing Basics

Scholarly productivity of participating faculty in the Research Development program was tracked pre- and post-completion using a publication tracking tool developed in-house by the New York University Health Sciences Library. This tool gathers citations to faculty publications from several online databases (e.g., MEDLINE, Embase, and Web of Science), into a single easy to use, searchable database. A report was generated for each participating faculty for which the search parameters were the year before participating in the Research Development program and during the year after completion, ranging from 2012 – 2020.

## Results

### Participation in research

Of the 29 students who responded to the 2021 annual research survey, 96.6% (n=28) indicated that they had research experience prior to LISOM. 27.6% (n=8) stated they were currently conducting extracurricular research with faculty, 58.6% (n=17) indicated they were interested in starting research with a faculty mentor, and 13.8% (n=4) stated they were not conducting research with faculty. The majority of research being conducted was clinical (87.5%, n=7), with quality improvement (25%, n=2) and epidemiology (12.5%, n=1) following [NB – this adds up to more than 100% because students could choose more than one type of research area]. Diverse departments were represented including radiology (12.5%, n=1), urology (12.5%, n=1), gynecology/obstetrics (25.0%, n=2), immunology (12.5%, n=1), psychiatry (12.5%, n=1), and pulmonology (12.5%, n=1). 88.5% of the projects were remote (n=7), with the rest conducted in the clinic (12.5%, n=1). Several projects involved COVID-19 research and most students (87.5%, n=7) reported their research schedules were flexible. Most students (87.5%, n=7) responded they were “very satisfied” with their research experience and 12.5% (n=1) responded that they were “neither very satisfied nor very dissatisfied”. Some free-form comments included that the students: “appreciate the flexibility with the research project so I am able to balance my other academic commitments”; they are “learning the knowledge and skills needed to conduct this kind of research in the future”; and they are “having a great educational experience in a low-stress environment” (see underlying data file 1 for student survey results) ^15^. One student reported applying for, and receiving, funding for their project. 50% of the students reported publishing their research and, of those, 50% were abstracts, 25% were papers, and 25% were book chapters.

Of the faculty research mentors who responded to the annual survey on satisfaction with research mentees (n=4 mentors out of n=11 total faculty responses), 75% (n=3) said they were “satisfied” or “very satisfied” and 25% (n=1) said they were neither “very satisfied” nor “very dissatisfied” with their experience mentoring medical students. Some faculty comments included: “[student was] eager, took initiative to learn new skills, we kept each other accountable for deadlines” and “excellent dedication from students!” The one faculty mentor who indicated they were “neither very satisfied nor very dissatisfied” commented that they “would have liked more contact” (see underlying data file 2 for faculty survey) ^15^.

For the faculty Researcher Pathway, although fewer faculty completed this curriculum compared to the Educator Pathway (n = 5 and n = 11, respectively), the quality of research projects deemed by the program director was higher than previous cohorts; research questions were clearer and more defined, there was less need for individual mentoring on basic concepts such as variable definitions and study designs, and reduced uncertainty among participants about data collection procedures. 

### Presentation of research

Of the students who reported conducting research (n=8), 37.5% said they presented their research at locations such as the institutional Annual Hospital Research Day and national conferences (e.g., American College of Rheumatology annual conference). According to data obtained from the leadership of the institutional Annual Hospital Research Day, this past year a total of 11 medical students presented their work, with two receiving awards of distinction.

For faculty, year 2021 was the first year participants from the Faculty Scholars Researcher Pathway were invited to submit their work to the Annual Hospital Research Day. According to conference leadership, a total of five faculty from this program presented their research. 

### Scholarly productivity

As of this writing, eight students have authored publications, six of them co-authored with NYU LISOM faculty. Additionally, of the faculty who completed the Research Development program throughout the years, 35% showed an increase in publications after completion.

## Discussion

### Summary of findings

Because of NYU LISOM’s accelerated nature, lack of time is a significant barrier to research for both students and faculty. Leadership at NYU LISOM appointed a faculty member to develop an extracurricular student research program, fulfilling the request for clinically-oriented research opportunities from students. This program helps connect interested students with faculty conducting research among diverse departments. Additionally, the program provides guidance on ethics training and holds biannual workshops on pertinent research-related skills. In conjunction, a faculty development research program had recently been revamped to train physicians in research methods promoting scholarly productivity, which is becoming a requirement at many institutions ^6– 10^. Our findings suggest that the synergy of these two programs contributed to successful collaborations between students and faculty. These new initiatives influenced the level of barriers to research engagement such as providing guidance to students on getting started, fostering connections between students and faculty interested in research, and providing opportunities for research with flexible scheduling, all leading to scholarly activity for both students and faculty.

Participation in research during medical school increases the likelihood of conducting research as a physician ^17^. Additionally, it helps students master skills that are important for future clinical practice including problem-solving, life-long learning, critical thinking, hypothesis formation, and communication ^17– 19^. Though undergraduate medical education research programs are successful at imparting necessary skills in future physicians, students feel that these programs should be voluntary, not mandatory ^19^. While students across the globe identify research as an important component of medical education, many find opportunities at their school lacking ^20^, highlighting the need for more programs promoting scholarship during undergraduate medical education.

### Limitations

The authors recognize limitations of this effort in that it only describes activity at one single institution. The student research program has only been in existence since 2020 so there is no data on how it impacts students’ literacy and competency in practicing evidence-based medicine after graduation. Further, since the student research program is voluntary, there are no mandatory reporting requirements other than ethics training and optional participation in annual surveys, so these data may not represent all students conducting research at this institution. Additionally, while many faculty indicated interest in conducting research with medical students, there were low response rates to annual faculty surveys (n=15 to the initial call for faculty and n=11 to the faculty satisfaction survey in 2021) which limits opportunities for both students and faculty.

### Future directions

While there are opportunities for students to conduct research at NYU LISOM among diverse departments, the leadership will begin preferentially promoting primary care-related research opportunities to align with the school’s mission: educating primary care physicians. Leadership will also emphasize the importance of faculty participation in annual research surveys at different venues. This process should help increase participation by faculty, opportunities for students, and promote productivity for students and faculty alike.

## Conclusion

The initiative described in this paper acknowledges and responds to the need for students and faculty in academic medicine to be well-versed in research skills and engage in collaborative relationships for scholarly activity. Overall, the outcomes of the student research program and faculty researcher pathway were positive in terms of student engagement in research and faculty scholarly productivity, though both programs are new and long-term outcomes are yet to be evaluated. Fellow institutions may look to the efforts described in this paper to build new program curricula for students and faculty targeting research development.

## Data availability

### Underlying data

Figshare. Fostering student and faculty scholarship in an accelerated three-year medical school. https://doi.org/10.6084/m9.figshare.19773166.v2 ^15^

This project contains the following underlying data:

- 2021 LISOM Student Research - results anonymized SUP DATA FILE 1.csv (anonymized results from students).
- 2021 LISOM Faculty Research Opportunities Satisfaction - results anonymized SUP DATA FILE 2.csv (anonymized results from faculty).

### Extended data

Figshare. Fostering student and faculty scholarship in an accelerated three-year medical school. https://doi.org/10.6084/m9.figshare.19773166.v2 ^15^. 

This project contains the following extended data:

- 2021 LISOM Student Research - SUP FIG2.pdf (blank English copy of the survey distributed to students)
- 2021 LISOM Student-Faculty Research – SUP FIG 1.pdf (blank English copy of the survey distributed to faculty)

Data are available under the terms of the Creative Commons Zero "No rights reserved" data waiver (CC0 1.0 Public domain dedication).
