# Supplementary material for: Fostering student and faculty scholarship in an accelerated three-year medical school
Source: MedEdPublish (2016). 2022 Jun 23;12:45. [Version 1] doi: 10.12688/mep.19142.1 (PMC9427079; doi:10.12688/mep.19142.1)
Supplement: Peer review history

# Peer review: Reviewer response for version 1

**DOI:** 10.21956/mep.20500.r32376

**Published:** 2022-08-15

**Authors:** Diann S. Eley

**Affiliations:** 1 Academy for Medical Education, The University of Queensland Medical School, Brisbane, Qld, Australia

Thank you for the opportunity to review this paper that reports on a program to enhance medical students’ and faculty members’ scholarly productivity by targeting research skills. 

- The paper is well written throughout and clear.
- The aims of the program are reasonable, but it appears that one big drawback is the shortened timeframe of the degree. This possibly means that students and staff will prioritise what they feel is most important. Making this program and extra-curricular activity sends a message that this is an add-on and certainly those who are not interested (yet) in research will treat it as such.
- I thought the outcomes were quite optimistic given the short timeframe to track published material from their study.
- I wonder why some sort of self-report on progress was not used even as an interim measure of success.
- I’m not sure I agree with the authors’ justification that research should only be done if one is passionate or interested in it. I do appreciate the School's shortened program and the time constraints and space for the inclusion of research skills alongside many other curricular requirements. However, I'm sure the authors did not mean to give the impression that research skills and an understanding of the nature of evidence-based practice were not important, and I would expect that some research skills were integrated into the curriculum.  
- The response rates for both students and faculty are probably reflecting time limitations. However, encouragement and a positive approach to getting involved in research and scholarly activities can be beneficial to raising interest.
- I am impressed with their efforts to continue the faculty programs. Often the faculty are more difficult to engage than the students. It's good that you have kept that going.

Overall, a worthwhile study hindered mainly by the impractical time constraints made worse, I would think, due to the shortened program.

Have any limitations of the research been acknowledged?

Partly

Is the study design appropriate and does the work have academic merit?

Yes

Is the work clearly and accurately presented and does it cite the current literature?

Yes

If applicable, is the statistical analysis and its interpretation appropriate?

Yes

Are all the source data underlying the results available to ensure full reproducibility?

No source data required

Are the conclusions drawn adequately supported by the results?

Yes

Are sufficient details of methods and analysis provided to allow replication by others?

Partly

Reviewer Expertise:

Medical education, personality and behaviour, career choice, rural medical workforce

I confirm that I have read this submission and believe that I have an appropriate level of expertise to confirm that it is of an acceptable scientific standard.

# Peer review: Reviewer response for version 1

**DOI:** 10.21956/mep.20500.r32266

**Published:** 2022-07-20

**Authors:** Alice Fornari

**Affiliations:** 1 Donald and Barbara Zucker School of Medicine at Hofstra/Northwell, Hempstead, NY, USA; 2 Northwell Health, New Hyde Park, NY, USA

Thank you for this thoughtful work on the scholarship of creating a research agenda in a new regional medical school with an accelerated curriculum that could limit research focus and productivity. This is a very important topic on the agenda of any medical school.

 I really like the harmony of doing parallel programs for students and faculty and also that it is extracurricular. I am unclear what the didactic content of the two programs was and maybe there can be an appendix to list the didactic preparatory topics. Did you ever consider joint classes for students and faculty and positioning for paired research projects resulting and joint accountability guiding them on their research journey? This seems to link the parallel process if I am understanding it correctly. In the future, you might consider a focus group with both sets of learners as survey response results are low. So, a mixed methods design for outcomes. Some added demographics on faculty would be informative too.

 Student demographic is provided. Also, with some success with faculty scholarship, I suggest you prepare a table for the appendices of exactly what the outcomes were with more detail to exemplify diversity and also support the notion of broadening scholarship beyond traditional profiles. Creating a culture of scholarship is not easy and you are trying with limited resources and assure flexibility and access to knowledge and skills. Look at your efforts as a culture shift for a new medical school where research is not the primary focus and acknowledge the need for primary care-based research as an important part of medical education across the continuum of learners. It cannot be ignored, and you are attempting to stir up interest to lead to desired outcomes. I suggest you also pick a program evaluation model, like Kirkpatrick as an example, to oversee your efforts and outcomes.

Have any limitations of the research been acknowledged?

Partly

Is the study design appropriate and does the work have academic merit?

Yes

Is the work clearly and accurately presented and does it cite the current literature?

Yes

If applicable, is the statistical analysis and its interpretation appropriate?

Not applicable

Are all the source data underlying the results available to ensure full reproducibility?

Yes

Are the conclusions drawn adequately supported by the results?

Yes

Are sufficient details of methods and analysis provided to allow replication by others?

Yes

Reviewer Expertise:

Mentoring, Teaching and Learning, Educational Scholarship, Health Humanities

I confirm that I have read this submission and believe that I have an appropriate level of expertise to confirm that it is of an acceptable scientific standard.
